# Understanding drivers of vaccine hesitancy among pregnant women in Nigeria: A longitudinal study

**DOI:** 10.1038/s41541-022-00489-7

**Published:** 2022-08-17

**Authors:** Gbadebo Collins Adeyanju, Philipp Sprengholz, Cornelia Betsch

**Affiliations:** 1 Psychology and Infectious Disease Lab (PIDI), Media and Communication Science, University of Erfurt, Erfurt, Germany; 2 Centre for Empirical Research in Economics and Behavioral Science (CEREB), University of Erfurt, Erfurt, Germany; 3 Bernhard Nocht Institute of Tropical Medicine (BNITM), Hamburg, Germany

**Keywords:** Population screening, Vaccines, Medical research, Infectious diseases

## Abstract

Vaccine-preventable-diseases are major contributors to disease burden in Sub-Saharan Africa. There is dearth of knowledge on the drivers of childhood vaccine hesitancy in Nigeria and its impact on coverage. Although understudied, pregnant women are a particularly important vulnerable group and very relevant for childhood vaccination decisions. This study’s aims are to adapt Confidence, Complacency, Constraints, Calculation, and Collective Responsibility, also known as the 5C psychological antecedence scale for the Nigerian context and to measure predictors of intention to vaccinate among pregnant women (prenatal) and subsequent vaccination behavior (postnatal). It is a longitudinal study that used multi-stage sampling procedure. One healthcare facility was selected from each district in five regional clusters, from which 255 pregnant women were randomly drawn. A standardized questionnaire was used to collect relevant data, including the 5C and some additional variables. Multiple linear regression using backward elimination analysis was performed to identify intention at prenatal and behavior at postnatal. Pregnant women’s intention to vaccinate unborn children was lower if they were Muslims, had lower confidence in public health system, if husband approval was important, and if they believed in rumor. At postnatal, vaccination behavior was more likely to follow mothers’ religious beliefs, when confidence in vaccine effectiveness was high and when mothers felt responsible for the collective. However, everyday stress (constraints) related to less vaccination behavior, and intention did not predict actual vaccination behavior. The 5C scale needs revision before being widely used in Nigeria. Yet, it is a better tool for measuring vaccination behavior than intention.

## Introduction

Since the new millennium, vaccine coverage for preventable infectious diseases in sub-Saharan Africa (SSA) has reached multiple targets, although acceptance and uptake still have the steepest height to climb in the region^1^. Even where successes have been recorded (increased vaccine uptake), the erosion of these gains owing to unclassified determinants or drivers and other vaccine-specific-related controversies in the region complicates responses and makes interventions even harder^2^. Unsurprisingly, 39 out of the 57 eligible countries for Global Alliance for Vaccines and Immunization (GAVI) vaccine support in 2020 were in SSA^3^. This will continue to plague the region if the determinants of vaccine hesitancy, especially in SSA, are not identified, studied, measured, and addressed^2^. Current knowledge on the gap and drivers of vaccine hesitancy in SSA is very scarce alongside knowledge about the extent of its impact on coverage or appropriate interventions^2^. This is partially because the design of interventions has been based on scientific research from high-income countries (HIC)^4,5^. Nevertheless, few interventions in SSA based on preliminary study outcomes have shown potency in improving vaccine acceptance^6^. To evaluate their real impact and effectiveness, it is important to test and validate measurement tools in SSA.

Parents, in general, and mothers, in particular, can strongly influence childhood vaccination decisions^7^. However, little is known about vaccine hesitancy among pregnant women. There is a likelihood that a mother’s hesitation could affect childhood vaccination^8–10^. This is partly why vaccine-preventable diseases account for more than one in eight child deaths before their fifth birthday in Nigeria^11^. In fact, Nigeria recorded an estimated average of 858,000 under-five deaths in 2019 alone^12^.

Reaching the Sustainable Development Goal (SDG) number 3 (Good Health and Well-being) by 2030 is becoming unlikely in Nigeria, in view of health outcome trends vis-a-vis the proportion of newborns^11,13,14^. According to Nigeria Demographic and Health Survey, the last two decades had seen a decline in under-five and infant mortality, however since 2016, under-five mortality is on the increase again from 120 deaths per 1000 live births in 2016 to 132 deaths per 1000 live births in 2018, while infant mortality has stopped declining and stagnant at 69 deaths per 1000 live births^11,13^. In 2019, Nigeria had an under-five mortality ratio of 201 per 1000 live births, meaning that one in five Nigerian children has not reached the age of 5, most of whom die of a vaccine-preventable disease^12,15^. At the same time, and despite the enormous financial resources expended on Expanded Program on Immunization (EPI), Nigeria is still one of the 10 countries in the world where 4.3 million children under five years old are without complete immunization^16^. The reasons for the surge in under-5 mortality and declining vaccine demand remain unclear. Shedding light on these general questions will be crucial. Specifically, it will be necessary to understand the drivers of vaccine hesitancy or enablers of low vaccine demand and to assess adapting the 5C model as a tool for measuring vaccine hesitancy in SSA/Nigeria context.

While substantial progress has been recorded globally on infant and child mortality by preventing infectious diseases, this has not been the same for most vulnerable populations, such as pregnant women and newborns through maternal immunization^17^. Studies have shown that more than 50% of the 2.76 million annual neonatal deaths globally are due to vaccine-preventable infections (22%) or pre-term births (35%), while about half of stillbirths are the resultant effects of maternal infections^18,19^. While child health has been improved substantially through vaccination over the years using EPI, little has been extended to other equally vulnerable groups, such as newborns and young infants^20^. As “vaccines are one of the most successful interventions to protect pregnant women and their fetuses, and infants from diseases that cause substantial morbidity and mortality”^17,21,22^, maternal immunization is beginning to receive increasing attention.

Pregnant women frequently refuse influenza (Flu) vaccines compared to Diphtheria, Tetanus, and Pertussis (DTP3)^23^. They and their babies are the most vulnerable groups because immunological and serological changes that occur during pregnancy can alter the mother’s and the fetus’s susceptibility to various infectious diseases^24^. Vaccinating pregnant women can have a double goal: to protect the mother from preventable diseases that affect her health and avoid infection/disease transmission to the fetus or newborn. Despite the potential benefits, pregnant women who doubt the real advantages of vaccines still resist vaccinations^25–29^.

Therefore, supporting maternal vaccination decision is critical during pregnancy because receiving vaccines generate maternal immune protection, transfers antibodies for early infant protection, and helps to assess the attitude during prenatal period^30^. Also, it is crucial to understand the drivers of the decision and its determinants. Therefore, the 5C psychological antecedent model are a means to do so. Furthermore, three additional variables were added to the model measuring drivers of vaccine hesitancy in Nigeria.

It is important to highlight the five main psychological antecedents of vaccination (5C) guiding this study. Since the analytical framework of this study was derived from the original 5C^5^, it is significant to buttress the measures. The measure goes beyond assessing confidence in the vaccine and the system administering the vaccines. Other relevant predictors are complacency (low-risk perception of vaccine-preventable diseases), constraints (structural and psychological barriers), calculation (meticulous search for vaccine information), and collective responsibility (consciousness to protect others)^5^. See Supplementary Table 1 for detailed conceptual definitions.

As validated by Betsch et al.^5^, confidence positively correlates with knowledge, attitudes, and trust in a health system. Complacency is expected to be negatively and positively related to risk perception of vaccine-preventable diseases and perception of good health status and invulnerability, respectively. Constraints correlate positively with a perception that everyday stress impedes vaccination action and a lack of self-control. Calculation is expected to have positive relations with a preference for critical thinking and well-informed decision-making. Collective responsibility correlates positively with communal orientation, collectivism-individualism, and empathy.

Besides exploring the validity of the 5C measure in SSA, specifically in Nigeria, three additional measures were considered as potential predictors such as religion, masculinity, and rumor/misinformation.

The primary goal is to adapt and validate the 5C scale for measuring vaccine hesitancy in Nigeria using two independent measurement points from the same participants (pre-post). Measurement point one during prenatal (T1) used the adapted scale to predict the vaccination intention of pregnant women. Measurement point two during postnatal (T2) was conducted 12 months postnatal to assess if intention (prenatal) translated into actual vaccination behavior. It is expected that the 5C will predict vaccination intention and behavior as described above. Moreover, we expected the three additional variables (religion, masculinity, and rumor/misinformation) to negatively influence vaccination intention and behavior^31,32^. Also, intention at T1 is expected to predict the mother’s vaccination behavior at T2.

## Results

### Demographic characteristics

At T1, 255 pregnant women participated in the study. Most respondents (63.9%) were between 25 and 34 years old. More than two-thirds of participants (67.8%) had tertiary education, while, overall, 96.5 and 2.4% had formal and informal education, respectively. Of 255 study participants, 69.4% were in their third pregnancy trimester, 22.7% were in their second trimester, and none in first. Participants from South-east are 24.4%, 12.4%—South-south, 16.5%—South-west, 19.8%—North-central, 16.9%—North-east, and 9.9%—North-west. Occupational statuses of participants’ distribution are as follows: 24.3% were public servants, 14.4%—employees of private entities, 36.2%—self-employed, 9.5%—unemployed, and 15.6%—housewives. Among the participants, 65.5% were mothers expecting their second or more child, and 32.2% were first-time mothers.

At T2, 96 women (representing 38%) included in the sample were 17–43 years old (with 64% between 24 and 34 years). 65.6% had tertiary education, and 70.1% were Christian. There seems to be not much difference between the distribution of participant characteristics at T1 and T2.

### Information sources and trust

The most significant vaccination information sources are during ante-natal care activities organized routinely at the hospitals by healthcare workers and during a consultation with doctors at 50.4 and 28.6%, respectively. Also, healthcare workers conducting antenatal care activities and doctors were the two most trusted sources for vaccination information by pregnant women at 87 and 82%, respectively.

### 5C antecedents of vaccination at T1

For all subscales of the 5C, reliability was low when compared to the original scale (see Appendix 1): Cronbach’s alpha was *α* = 0.41 for confidence, *α* = 0.32 for complacency, *α* = 0.45 for constraints, *α* = 0.51 for calculation, and *α* = 0.26 for collective responsibility. These coefficients were low compared to those obtained in Western samples, which were 0.71 and higher^5^. An additional explorative factor analysis did not reveal an alternative factor structure. Consequently, the 15 items of the 5C scale could not be aggregated. Instead, subsequent analyses were performed using the single items. Table 1 describes the 5C, including the extensions rumor, religion, and masculinity.

**Table 1:** Descriptive of 5C+ at T1 and T2.

	T1		T2	
	*M*	*SD*	*M*	*SD*
Confidence				
Vaccines are safe	4.53	1.08	4.91	0.41
Vaccinations are effective	4.45	1.24	4.81	0.56
Public authorities decide in the best interest of the community	3.62	1.58	4.70	0.79
Complacency				
Vaccination-preventable diseases are not common anymore	1.58	1.22	1.35	1.08
The immune system protects against diseases	2.69	1.71	4.23	1.37
Vaccine-preventable diseases are not severe	1.50	1.19	1.23	0.62
Constraints				
Everyday stress prevents vaccination	1.63	1.15	1.43	1.06
Receiving vaccinations is inconvenient	1.99	1.55	1.50	1.18
Visiting the doctor’s makes me feel uncomfortable	1.29	0.90	1.16	0.51
Calculation				
Weighing benefits and risks	3.63	1.67	3.32	1.82
Considering usefulness of vaccination	4.01	1.54	3.39	1.80
Vaccination topic must be fully understood	3.51	1.67	4.48	1.18
Collective Responsibility				
No need for vaccination when everyone is vaccinated	1.37	1.03	1.13	0.39
Vaccination to protect people with a weaker immune system	3.71	1.63	3.57	1.75
Vaccination as collective action to prevent the diseases’ spread	4.47	1.25	4.91	0.39
Religion				
Religion does not support vaccination	1.26	0.89	3.89	1.69
Masculinity				
Husband’s approval important for vaccination	1.26	0.79	3.14	1.74
Rumor				
Believing that vaccination causes infertility	1.94	1.53		
Rumors				
Belief in rumors (mean score)			2.33	0.97

*Note*. The rumor for T2 is average score. *M* mean value. *SD* standard deviation.

### Determinants of the intention to vaccinate child at T1

The analysis was performed to determine the demographic and psychological variables related to routine vaccination intention at T1. Age, religion, education, employment, the 5C, masculinity (father’s attitude), believing in rumors/misinformation (i.e., that vaccination can cause infertility), and thinking that one’s religion is incompatible with religious beliefs were considered predictors of the intention to vaccinate. Table 2 shows the regression results. Only four significant predictors were found. Muslims indicated lower vaccination intentions compared to Christian participants. Vaccination intentions increased with confidence in the public authorities/health system but decreased if participants indicated that their husband’s approval was important for vaccination and if they believed in the rumor that vaccination causes infertility.

**Table 2:** Regression results (T1).

Predictor	*β*	*b*	CI−	CI+
(Constant)		6.72	6.22	7.23
Being Muslim (vs. being Christian)	**−0.16**	**−0.29**	−0.58	−0.01
Public authorities decide in the best interest of the community	**0.24**	**0.12**	0.04	0.21
Everyday stress prevents vaccination	0.12	0.10	−0.03	0.23
Receiving vaccinations is inconvenient	0.15	0.08	−0.02	0.19
Visiting the doctor’s makes me feel uncomfortable	0.14	0.17	−0.03	0.36
Husband’s approval important for vaccination	**−0.21**	**−0.20**	−0.38	−0.02
Thinking that religious influence is a significant determinant of vaccine refusal	−0.17	−0.09	−0.17	0.00
Believing that vaccination causes infertility	**−0.26**	**−0.21**	−0.33	−0.08

*Note*. Results from the analysis (predictors: age, religion, education, employment, the 5C+, husbands’ approval important for vaccination, believing in a certain rumor and religious influence). *R*^2^ = 0.219, adjusted *R*^2^ = 0.171. Bold values are significant with *p* < 0.05. CI− and CI+ are the lower and upper bounds of the 95% confidence interval. *β* = beta.

### Determinants of actual vaccination behavior (T2)

At T2, the analysis controlled for T1 vaccination intention. The analysis used postnatal 5C, masculinity, compatibility of religion, and belief in vaccination being used for population control (rumor) to predict the vaccination behavior (Table 3). The results revealed that vaccination was more likely when it followed mothers’ religious beliefs, when confidence in vaccine effectiveness was high, and when mothers felt responsible for the collective (collective responsibility). Conversely, a higher level of everyday stress (constraint) was related to less vaccination behavior. Interestingly, prenatal vaccination intentions (T1) did not play a role in actual vaccination behavior (T2).

**Table 3:** Regression results at T2.

Predictor (as measured at T1)	*β*	*b*	CI−	CI+
(Constant)		0.49	0.07	0.92
Vaccinating my child goes along with my religious belief	**0.39**	**0.05**	0.03	0.07
Vaccination is an effective way to prevent disease	**0.21**	**0.08**	0.02	0.15
Stress prevents me from child vaccination	**−0.28**	**−0.07**	−0.11	−0.03
When everyone is vaccinated, I do not have to vaccinate my child	**−0.31**	**−0.18**	−0.27	−0.08
For each and every vaccination I consider if it is useful	0.13	0.02	0.00	0.04

*Note*. Results from the analysis (predictors: 5C, husbands’ approval, compatibility of religion, and belief in vaccination being used for population control). *R*^2^ = 0.58, adjusted *R*^2^ = 0.56. Bold values are significant with *p* < 0.05. CI− and CI+ are the lower and upper bonds of the 95% confidence interval. *β* = beta.

More analyses were conducted to explore the correlates of the four significant behavioral predictors identified above. Demographic variables (age and education) and belief in various conspiracy theories were entered as predictors (Table 4). Age was unrelated to any of the variables. Higher education strengthened the belief in vaccination effectiveness and collective responsibility. The belief in rumors and misinformation was related to all variables.

**Table 4:** Regression results.

	Vaccinating my child goes along with my religious belief				Vaccination is an effective way to prevent disease				Stress prevents me from child vaccination				When everyone is vaccinated, I do not have to vaccinate my child			
Predictor	*β*	*B*	CI−	CI+		*b*	CI−	CI+		*b*	CI−	CI+	*β*	*b*	CI−	CI+
(Constant)		**3.03**	2.20	3.87		**4.51**	3.86	5.16		**0.91**	0.55	1.28		**2.13**	1.73	2.53
Education: primary (Baseline: no education)					0.25	0.63	−0.13	1.39					**−0.63**	**−1.06**	−1.53	−0.60
Education: secondary (Baseline: no education)					0.33	0.48	−0.18	1.13					**−1.15**	**−1.11**	−1.50	−0.71
Education: tertiary (Baseline: no education)					**0.56**	**0.71**	0.09	1.33					**−1.40**	**−1.20**	−1.58	−0.82
Vaccination is a means of population control	**−0.25**	**−0.30**	−0.53	−0.07					**0.38**	**0.32**	0.15	0.49				
Children get sick of spiritual attacks	**0.23**	**0.25**	0.03	0.46												
Prayers are an effective way to prevent diseases	**0.31**	**0.30**	0.10	0.50												
I doubt the sincerity of countries providing vaccines					**−0.39**	**−0.17**	−0.25	−0.08					**0.22**	**0.07**	0.02	0.12
*R*^2^/adjusted *R*^2^		0.30/0.27			0.24/0.21				0.15/0.14				0.40/0.37			

*Note*. Results from the analysis (predictors: age, education, conspiracy theories, and rumors from the table). Bold values are significant with *p* < .05. CI− and CI+ are the lower and upper bonds of the 95% confidence interval. *β* = beta.

## Discussion

Assessment of the 5C model for predicting prenatal intention and postnatal vaccination behavior is a novel contribution to the body of knowledge for addressing vaccination demand in Nigeria. The primary goal was adapting and validating the 5C scale using two independent measurement points from the same subjects (pre-post), where T1 assessed the vaccination intention of pregnant women at prenatal, and T2 considered actual behavior and decision months after childbirth at postnatal. Adapting the scale to predict vaccination intention and behavior in Nigeria was novel and revealing.

Reliability analyses for the 5C based on the original model and sequel upon examination were incompatible in the Nigerian setting. The internal consistency indicators were too low for all five indicators as compared to reliability indicators obtained in Western samples. This shows that the items did not fit well with their intended constructs in Nigeria as compared to the Western samples. Therefore, the assumption that the 5C scale or factors that predict vaccine hesitancy was wholly adaptable and valid in Nigeria was undetermined by this study. Nevertheless, items from the scale predicted behavior (more than intention) and additions to the scale proved useful in the Nigerian context.

Higher confidence as assessed after birth was related to better vaccine uptake. The measurement of the psychological antecedent of confidence seems to be the strongest predictor of the 5C scale, especially the item that measures confidence in the public authority/health system. The study shows that participants’ confidence in the country’s healthcare system is related with a more positive intention to vaccinate their child in the future. It may be assumed that the less mothers interact with and are exposed to vaccination knowledge from healthcare providers, healthcare workers, or public institutions managing vaccination, the lower their intention to vaccinate children, and vice versa. Confidence among mothers correlates with the assurance that vaccinating their unborn children would be very low in situations of a lack of trust in the healthcare workers and systems managing vaccination decisions in the country. This evidence could explain the slow, stagnant, and/or declining childhood vaccination rates, which peaked at 31% in 2018 against the 90% target, and the continued high rate of neonatal, under-five, and infant mortality in Nigeria^11,13^. Generally, there is declining confidence in Nigeria’s public authority/health system, especially for immunization, family planning, antenatal care, etc.^11,13,14^. This lays credence to why Nigeria remains the country with both the highest under- and unvaccinated children in the world in 2018^33–36^.

The confidence-eroding factor in the public authority or health system here could also be linked to misconceptions about the preventive role of immunization in Nigeria, especially where uptake rates are very low. Studies found that some healthcare workers and health system managers exaggerate about immunization to motivate uptake, thereby giving caregivers the false impression that immunization prevents all childhood diseases ^37,38^. Hence, the inability or failure of immunization to prevent all diseases erodes trust and confidence in the public institutions managing immunization and eventually the loss of faith in immunization as an intervention to give protection^37^.

Also, the source of information and communication is critical to the perception developed by people toward vaccination. Based on this study, mothers who received their vaccination information through the antenatal care services/healthcare workers and doctors are confident about vaccine efficacy, are more likely to have a positive attitude toward vaccination, and have a higher intention to vaccinate their children. Therefore, more should be done to link antenatal care services to all primary healthcare facilities in communities to enhance the knowledge required for healthy and safe childbirth, thereby increasing vaccination demand.

Another important finding was the role of religion. Religion significantly influences decision-making in many parts of SSA. Studies including the Pew survey found religion to be central to discourse about behavior and decision-making in SSA^39–42^. The confirmation of religion as a determinant of childhood vaccination intention among mothers was therefore not surprising. The variation among religious groups is even more affirmative, where Muslim mothers have a lower motivation toward vaccination than Christians. This behavior paralleled other studies that have examined the back-end impacts of religion or religious belief on vaccine uptake, either in SSA or Nigeria^43^. It permeates individual, group, and national decision-making, and the immunization system is no exception.

Regions with strong Islamic influence have lower immunization coverage and high vaccine hesitancy, adding to other variables such as low literacy levels^44–46^. The Muslim-dominated northern region, for example, has the highest vaccine hesitancy level, demonstrated by being the region with lowest immunization coverage: the least being North-West at 8% and the highest being North-Central at 26%^13,37^. Hence, a religiously biased targeted intervention approach is required. Since trust in vaccination information from antenatal care was highest, Nigeria’s immunization stakeholders should integrate “religious talk on vaccine acceptance” into their activities. Also, enlisting the support of influential Muslim women organizations such as the Federation of Muslim Women Association of Nigeria (FOMWAN) could be strategic.

In several communities in the SSA a strong patriarchal culture subsists^47^. Therefore, the child’s father or husband’s opinion is crucial in overall household vaccination decision-making. When a husband’s or child’s father’s attitude is relevant for the vaccination decision, vaccination was less likely. Other studies also found that children of women who lack decision-making autonomy are less likely to complete childhood immunization^48,49^. Therefore, strengthening fathers’ trust in and approval of vaccination will likely support the willingness of mothers to protect their children’s health by vaccinating them.

The data collection at T2 was critical to understand what predicts actual vaccination behavior (vaccination decision 12 months after childbirth). As observed in the analyses, vaccination intention was not meaningfully related to actual behavior. However, vaccination was related to three of the 5C: when confidence in the vaccine effectiveness was high (Confidence), when mothers felt responsible for collective well-being (Collective responsibility), and when mothers indicated lower levels of everyday stress (Constraint). Likewise, additional variables (5C+) played an additional role in vaccination behavior. Mothers of infants were more likely to vaccinate their children when vaccination was supported by the mothers’ religious beliefs (Religion). Rumor and/or misinformation was a very powerful influencing factor among all four predictors of vaccination behavior. That is, misinformation or rumor about vaccination affects these four determinants and thus indirectly affects vaccination behavior. Rumor and/or misinformation about vaccination should therefore be addressed with effective rebuttal strategies^50,51^.

Constraints such as stress arising from the mother’s competing priorities that displaces vaccination or contributed to miss appointments must be isolated and addressed. Facilitating or incentivizing vaccination visits could be an option. Increasing the resources (human and logistics) for home visits will allay the barriers that make vaccination very stressful for mothers.

Also, since the study revealed that mothers who possess higher education were related to stronger beliefs in vaccination effectiveness and collective responsibility, mothers of infants with lower education should be a target group for vaccination literacy campaigns.

The study had some limitations that might have affected the outcomes. Based on observations during the fieldwork, the 5C scale did not produce the desired effect. We can only speculate about the reasons. Its constructs and coinage of the single items may misfit the Nigerian cultural setting. More so, attempts to brief a couple of participants on the meaning of some items for clarity may have affected interpretations. Also, participants with low literacy (i.e., mothers with no or little education) struggled to fully understand the English grammar of some of the 5C items. Therefore, the 5C scale in the future needs to be rewritten in simpler and loose English grammar. Also, assessing and including pregnant women in the study takes a lot of logistics, despite targeting antenatal meetings.

In conclusion, the significance of confidence, constraints, collective responsibility, religion, rumors or misinformation, and masculinity as drivers of vaccination demand were a big step towards increasing the body of knowledge on vaccine hesitancy and its determinants, especially among vulnerable and understudied groups such as pregnant women in Nigeria. Second, the unexpected findings that prenatal vaccination intentions do not predict actual vaccination behavior and that confidence in effectiveness of vaccines was not a significant predictor at T1 but at T2, might show that, during pregnancy, vaccination is not a priority or a relevant issue, rather the decision about such an important undertaking is undecided at prenatal. I.e., the search for information or knowledge for vaccination decision-making takes place later when the child is born.

Although, the 5C scale did not provide the outcome as was obtainable in the western setting, it was a good model for understanding how intention and behavior interact with vaccination decision-making. At best, it is a valuable tool for understanding vaccination demand. Also, fathers of children eligible for vaccination and husbands are key target groups for national vaccination demand creation. Their vaccination decision-making authorities in households in the study setting is very crucial. An evidence-based intervention similar to those currently being used for family planning and antenatal programs would be good models.

Further studies are still necessary to align a suitable scale for measuring vaccine hesitancy in the SSA sub-region and Nigeria in particular. There is a need to increase empirical studies that ventured into understanding behavioral insights surrounding vaccine hesitancy in SSA. Even more dire are studies that focus specifically on pregnant women’s childhood vaccination decision-making process. Future studies might consider emergence of a new scale for measuring vaccine hesitancy using items from confidence, constraints, collective responsibility, religion, rumor, and masculinity.

## Methods

The study was conducted according to the guidelines of the Declaration of Helsinki and approved by the Institutional Review Board (Ethics Committee) of National Health Research Ethics Committee in Nigeria for both T1 and T2 studies (reference number FHREC/2018/01/99/03-09-2018/19). Written informed consent was obtained from all participants. The default language of the study was English since this is the official language. Although, other languages are also widely spoken.

### Study design and setting

In this study, we used a longitudinal study design with a multistage sampling procedure at T1. A convenient sample of primary units was obtained from five clusters (geographical zones), and then a sample of secondary units was selected from each primary unit^52^. Nigeria’s capital territory (Abuja) was divided into five clusters. One healthcare facility was randomly selected from each cluster, from which participants (pregnant women attending antenatal check-ups) were randomly drawn. Out of the five health facilities, two were primary health centers, one was a general hospital, one was a tertiary health facility/specialist hospital, and the last was a private hospital. Data collection at T1 occurred in the fall of 2018. For T2, all participants who provided their contact details were directly contacted via telephone and included upon consent. Data collection at T2 took place in the fall of 2019.

### Participants

At T1, participants were pregnant women attending routine antenatal care. The rationale for this focus was based on a) the primary outcome of the study, which was the mothers’ intention and decision to vaccinate her child, and b) the morbidity and mortality from the vaccine-preventable disease being highest among children under five (U5M) in the country of study^53^. Therefore, eligibility for inclusion was being female and pregnant. Participants during the prenatal study gave consent to be included and contacted for follow-up study at T2. Upon invitation for the postnatal study, 96 participants agreed to participate out of the 179 successfully contacted. Out of the 96 participants, the ages of their children ranged from six to 12 months old.

### Measures at T1

A structured questionnaire was developed using the 5C+ scale. Participants filled in the items for demographic information, 5C+, vaccination intention, and trust in sources of vaccination information. The items for each scale were randomized (within scale) before printing the questionnaires and remained in the same order for all participants. Upon request, some participants were briefed on the meaning of some items for clarification.

On the *demographic characteristics*, the study assessed age, gender (female, male, and other), education attainment (primary, secondary, tertiary, others), region of origin (South-West, South-East, South-South, North-West, North-East, and North-Central), type of employment (public service, self-employed, unemployed, private company/NGO, and housewife), marital status/married (yes, no, have a partner), and religion (Christian, Muslim, traditional religion, no religion).

On the *5C psychological antecedents of vaccination and additional variables (5C*+*)*, the 5C scale comprised 15 items as reliable indicators of confidence, complacency, constraints, calculation, and collective responsibility. Five of them represented a short version with one item per construct. *Confidence*: “I am completely confident that vaccines are safe.” “Vaccinations are effective.” “Regarding childhood vaccines, I am confident that public authorities decide in the best interests of the community.” *Complacency:* “Vaccination is unnecessary because vaccine-preventable diseases are not common anymore.” “My immune system is so strong that it also protects me against diseases.” “Vaccine-preventable diseases are not so severe that I should get vaccinated.” *Constraints:* “Everyday stress prevents me from getting vaccinated.” “For me, it is inconvenient to receive a vaccination.” “Visiting the doctors makes me feel uncomfortable; this keeps me from getting vaccinated.” *Calculation:* “When I think about getting vaccinated, I weigh the benefits and risks to make the best decision possible.” “For each and every vaccination, I closely consider whether it is useful for me.” “It is important for me to fully understand the topic of vaccination before I get vaccinated.” *Collective Responsibility:* “When everyone is vaccinated, I do not have to get vaccinated too.” “I get vaccinated because I can also protect people with weaker immune system.” “Vaccination is a collective action to prevent diseases’ spread.” The response scale ranging from 1 = strongly disagree to 5 = strongly agree was used.

The additional Variables (5C+) were religion, masculinity, and rumor. We refer to the adapted version as 5C+. *Religion* is another important aspect of everyday life that affects people’s attitudes toward vaccination^54–56^. Therefore, we included “My religion is incompatible with vaccination, so I may refuse immunization for my child” (1 = strongly disagree to 5 = strongly agree). *Masculinity* variable measured the extent of the influence of fathers or husbands on childhood vaccination decisions in households. Their attitudes toward vaccination play an important role in acceptance or refusal^57–59^. Mothers who solely depend on their husbands’ or child’s father’s approval are susceptible to vaccine hesitancy if he refuses^60^. The item was “What is your spouse’s view on vaccination?” (1 = strongly discourage vaccination to 5 = strongly encourage). This item intended to assess whether the husband or the child’s father has a say in the childhood vaccination decision. The need for father’s approval of vaccination is often cited as a challenge for vaccine uptake in SSA^61–64^. As there were many questions of the participants regarding the meaning of the question and as the wording missed the intended meaning at T1, it was rephrased at T2. *Rumor/misinformation* affects the perception and attitude of people, especially as social media continues to dominate the amplification of vaccine misinformation, thereby affecting vaccination demand^31,32,65^. It makes the inclusion even more relevant. The variable assessed conspiracy belief such as “Immunization causes infertility” (1 = strongly disagree to 5 = strongly agree).

*Information sources and trust* such as sources of vaccination information and participants’ levels of trust in each of these sources were measured. The sources included antenatal sessions at the health facility; social media; family and friends; church or mosque; and doctor’s consultations. Trust in these sources of information was assessed using a scale of 1–7: 1 = do not trust at all and 7 = I totally trust.

*Vaccination intention* was measured by a single item: “How will you decide on your new baby’s first vaccination?” (1 = definitely not vaccinate, 5 = definitely vaccinate).

### Measures at T2

*Vaccination behavior* measurement instruments are the same as at T1, except that the main dependent variable was actual vaccination behavior that had taken place until at T2, i.e., vaccines received by children within the period and corresponding to the routine immunization recommendation. Behavior was assessed as “Has your youngest child received the following recommended vaccines (BCG, Polio, Hepatitis B, Yellow Fever, and DTP3)?”. The vaccination cards of all included participants were sighted for confirmation.

*Religion, rumor/misinformation and masculinity* were equally measured. At T2, religion was measured as “Vaccinating my child goes along with my religious beliefs” (1 = strongly disagree to 5 = strongly agree). Based on feedback from participants at T1, the religious belief question was rephrased into simple language at T2 for proper understanding. For rumor/misinformation, items assessed included “Vaccination is a means to control population growth in Nigeria” “I doubt the sincerity of the countries providing the vaccines” “Prayers are an effective way to prevent measles” and “Children become sick because of spiritual attacks or witchcraft” (1 = strongly disagree to 5 = strongly agree). While for masculinity, “My husband’s approval is important in order to vaccinate my child” (1 = strongly disagree to 5 = strongly agree).

### Data analysis

The data were analyzed using R (version 3.6.3). To determine the reliability of the 5C subscales, Cronbach’s α was calculated. However, due to poor reliability, multiple linear regression model with backward elimination analysis of all items was used to identify determinants of pregnant women’s vaccination intention. Demographic variables, all 5C+ items, belief in rumors and misinformation, and trust in different information sources were considered as predictors. For vaccination behavior at T2, the same analysis was performed. Vaccination behavior was calculated as the mean of BCG, Polio, Hepatitis B, Yellow Fever, and DTP3 vaccinations. Each participant received a score = 1 if some or all the recommended doses were administered and 0 if none of the doses was given. If no information about some doses was provided, the vaccination was not considered in the mean calculation.

### Reporting summary

Further information on research design is available in the Nature Research Reporting Summary linked to this article.

## Supplementary information

Supplementary information

REPORTING SUMMARY

## Data Availability

The datasets generated and/or analyzed during the current study are available at https://osf.io/3k9n8/.
