# Supplementary material for: Understanding drivers of vaccine hesitancy among pregnant women in Nigeria: A longitudinal study
Source: NPJ Vaccines. 2022 Aug 17;7:96. doi: 10.1038/s41541-022-00489-7 (PMC9385635; doi:10.1038/s41541-022-00489-7)
Supplement: Supplementary file 1 — Supplementary information [file 41541_2022_489_MOESM1_ESM.pdf]

Supplementary Information

Supplementary Information 1

Understanding Concepts of the 5C Psychological Antecedence [5].

Confidence	*Confidence* "is defined as trust in the effectiveness and safety of vaccines, the system that delivers them, including the reliability and competence of the health services and health professionals, and the motivations of policy-makers who decide on the need of vaccines". Individuals who lack confidence have negative attitudes toward vaccination.
Complacency	*Complacency* "exists where perceived risks of vaccine-preventable diseases are low, and vaccination is not deemed a necessary preventive action." Complacent individuals do not feel threatened by infectious diseases and thus have no impetus to change their prevention behavior. Complacency is usually negatively related to the perceived risks of diseases.
Constraints	*Constraints* are issues when "physical availability, affordability and willingness-to-pay, geographical accessibility, ability to understand (language and health literacy) and appeal of immunization service affect uptake." In this study, we expected positive correlations with perceived time pressure and daily hassles and a negative correlation with perceived access to healthcare.
Calculation	*Calculation refers* to individuals' engagement in extensive information searching. We assumed that individuals high in calculation evaluate the risks of infections and vaccination to derive a good decision.
Collective Responsibility	C*ollective responsibility* is defined as the willingness to protect others by one's own vaccination through herd immunity. Thus, people high in collective responsibility are willing to vaccinate in another person's or community's interest. Conversely, having low values indicates that a person does not know about herd immunity, does not care or want to vaccinate for others' benefit.
