# Supplementary material for: Understanding drivers of vaccine hesitancy among pregnant women in Nigeria: A longitudinal study
Source: NPJ Vaccines. 2022 Aug 17;7:96. doi: 10.1038/s41541-022-00489-7 (PMC9385635; doi:10.1038/s41541-022-00489-7)
Supplement: Supplementary file 2 — REPORTING SUMMARY [file 41541_2022_489_MOESM2_ESM.pdf]

nature portfolio

Corresponding author(s): Gbadebo CollinsAdeyanju

Last updated by author(s): Mar 21, 2020

Reporting Summary

Nature Portfolio wishes to improve the reproducibility of the work that we publish. This form provides structure for consistency and transparency in reporting. For further information on Nature Portfolio policies, see our Editorial Policies and the Editorial Policy Checklist.

Statistics

For all statistical analyses, confirm that the following items are present in the figure legend, table legend, main text, or Methods section.

n/a	Confirmed	
☐	☒	The exact sample size (*n*) for each experimental group/condition, given as a discrete number and unit of measurement
☐	☒	A statement on whether measurements were taken from distinct samples or whether the same sample was measured repeatedly
☐	☒	The statistical test(s) used AND whether they are one- or two-sided<br>*Only common tests should be described solely by name; describe more complex techniques in the Methods section.*
☐	☒	A description of all covariates tested
☐	☒	A description of any assumptions or corrections, such as tests of normality and adjustment for multiple comparisons
☐	☒	A full description of the statistical parameters including central tendency (e.g. means) or other basic estimates (e.g. regression coefficient) AND variation (e.g. standard deviation) or associated estimates of uncertainty (e.g. confidence intervals)
☒	☐	For null hypothesis testing, the test statistic (e.g. *F*, *t*, *r*) with confidence intervals, effect sizes, degrees of freedom and *P* value noted<br>*Give P values as exact values whenever suitable.*
☒	☐	For Bayesian analysis, information on the choice of priors and Markov chain Monte Carlo settings
☒	☐	For hierarchical and complex designs, identification of the appropriate level for tests and full reporting of outcomes
☒	☐	Estimates of effect sizes (e.g. Cohen's *d*, Pearson's *r*), indicating how they were calculated

Our web collection on statistics for biologists contains articles on many of the points above.

Software and code

Policy information about availability of computer code

Data collection	No software was used
Data analysis	R software (version 3.6.3).

For manuscripts utilizing custom algorithms or software that are central to the research but not yet described in published literature, software must be made available to editors and reviewers. We strongly encourage code deposition in a community repository (e.g. GitHub). See the Nature Portfolio guidelines for submitting code & software for further information.

Data

Policy information about availability of data

All manuscripts must include a data availability statement. This statement should provide the following information, where applicable:

- Accession codes, unique identifiers, or web links for publicly available datasets
- A description of any restrictions on data availability
- For clinical datasets or third party data, please ensure that the statement adheres to our policy

The datasets generated and/or analyzed during this study are available in https://osf.io/3k9n8/

Field-specific reporting

Please select the one below that is the best fit for your research. If you are not sure, read the appropriate sections before making your selection.

☐ Life sciences ☒ Behavioural & social sciences ☐ Ecological, evolutionary & environmental sciences

For a reference copy of the document with all sections, see nature.com/documents/nr-reporting-summary-flat.pdf

Behavioural & social sciences study design

All studies must disclose on these points even when the disclosure is negative.

Study description	This is a longitudinal study and used quantitative data.
Research sample	The sample are pregnant women in the federal capital territory of Nigeria. Sample are included if pregnant and above 18 years old. The sample is convenient. The reason is because, the population is very large and there is paucity of funds to be able to logistically cover it. Also, the sample are the most vulnerable among adult population and most difficult to access.
Sampling strategy	The participants were randomly chosen based on districts stratification (north, south, east and west) within the population. The rationale for the sample size was based on estimated judgement and saturation of access to pregnant women in within the population. Since it is a longitudinal study, the sample size was sufficient to test the study goal...whether vaccination intention leads to actual behavior and if the 5C is a good instrument to achive it.
Data collection	The traditional questionnaire using paper and pencil was used to collect data. Each participant was given the printed questionnaire and a pencil to choose answer (s) that best represent their views. It was done anonymously and unaided, within the viewing distance of the researcher.
Timing	The first data collection was done between 26. 09. – 25.10.2018, while the second on same sample was done between 27.08 – 26.09.2019.
Data exclusions	No data excluded
Non-participation	In the second data collection phase of same sample, some participants dropped out because they have moved and others because of time. While others noted privacy reasons.
Randomization	Participants were randomly selected, no allocation.

Reporting for specific materials, systems and methods

We require information from authors about some types of materials, experimental systems and methods used in many studies. Here, indicate whether each material, system or method listed is relevant to your study. If you are not sure if a list item applies to your research, read the appropriate section before selecting a response.

Materials & experimental systems

n/a	Involved in the study	
☒	☐	Antibodies
☒	☐	Eukaryotic cell lines
☒	☐	Palaeontology and archaeology
☒	☐	Animals and other organisms
☐	☒	Human research participants
☒	☐	Clinical data
☒	☐	Dual use research of concern

Methods

n/a	Involved in the study	
☒	☐	ChIP-seq
☒	☐	Flow cytometry
☒	☐	MRI-based neuroimaging

Human research participants

Policy information about studies involving human research participants

Population characteristics	See above
Recruitment	See above
Ethics oversight	Nigeria Health Research Ethics Committee

Note that full information on the approval of the study protocol must also be provided in the manuscript.
